# Roles of Single Nucleotide Polymorphisms in the SLC6A2 Gene in the Risk of Vasovagal Syncope Among Children in Eastern China: A Case‐Control Study

**DOI:** 10.1002/hsr2.70585

**Published:** 2025-03-19

**Authors:** Minmin Wang, Meng Li, Haizhao Zhao, Xiaoyue Liu, Qingyu Kong, Cuifen Zhao, Baomin Li

**Affiliations:** ^1^ Department of Pediatrics Qilu Hospital of Shandong University Jinan Shandong China

**Keywords:** children, etiology, SLC6A2, vasovagal syncope

## Abstract

**Background and Aims:**

Vasovagal syncope (VVS) is a primary reason for fainting in children, affected by numerous genetic and environmental factors. We designed this study to investigate the impact of SLC6A2 gene polymorphisms and gene‐environment interactions on the etiology of VVS in children.

**Methods:**

This study was conducted with 142 children, comprising 71 VVS patients and 71 healthy controls. Five single nucleotide polymorphisms (SNPs) in the SLC6A2 gene (rs2242446, rs168924, rs2397771, rs5564, and rs5569) were genotyped using TaqMan assays. Clinical and hematological data were analyzed alongside genetic information.

**Results:**

The rs2242446 TT genotype was significantly associated with VVS (CC + CT/TT, 31/40 vs. 45/26, *χ* ^2^ = 5.55, *p* = 0.02), and the AA genotype of rs5564 was similarly correlated with increased VVS risk (GG + AG/AA, 39/32 vs. 27/44, *χ* ^2^ = 4.08, *p* = 0.04). No significant associations were identified for rs168924, rs2397771, or rs5569. Additional risk factors include family history, elevated hemoglobin (HB) concentrations, increased mean corpuscular volume (MCV), and low vitamin D levels. Multivariate analysis showed that only rs2242446 remained significantly associated with VVS (CC + CT/TT; OR, 2.54; 95% CI; 1.12–5.75; *p* = 0.03). Vitamin D deficiency, family history, and certain hematological markers were also notable risk factors.

**Conclusion:**

Polymorphisms in the SLC6A2 gene, particularly rs2242446, may increase the risk of VVS in children. Further research is needed to validate these findings and explore therapeutic interventions targeting the norepinephrine system.

## 1 Introduction

Vasovagal syncope (VVS) is the predominant contributor to fainting in children, amounting to 60% to 80% of cases [1]. It is marked by sudden drops in heart rate and blood pressure, leading to reduced cerebral blood flow and temporary loss of consciousness [2]. Though often benign, recurrent VVS significantly impacts young patients' quality of life, contributing to both physical and psychological issues such as anxiety and fear, and it can create emotional burdens for families [3]. The pathophysiology of VVS is complex, involving mechanisms such as the Bezold‐Jarisch reflex, autonomic dysfunction, neurohumoral factors, vascular influences, and gut microbiota alterations [4]. Elevated plasma levels of epinephrine and norepinephrine (NE) before and during syncope episodes suggest dysregulation of the sympathetic nervous system [5].

The norepinephrine transporter (NET), encoded by the SLC6A2 gene, is key in NE reuptake from the synaptic cleft, thereby maintaining cardiovascular stability. Genetic variations in SLC6A2, particularly single nucleotide polymorphisms (SNPs), have been linked to cardiovascular and psychiatric conditions such as cardiovascular response to drug, depressive disorder, and attention‐deficit hyperactivity disorder (ADHD) [6, 7, 8, 9, 10]. These genetic variations complicate the genotype‐phenotype relationship due to their impact across both cardiovascular and psychiatric domains. Polymorphisms in genes like SLC6A2 influence neurotransmitter regulation, behavior, and disease traits, highlighting the complexity of these associations [11]. Refining phenotypes and considering environmental factors, especially early‐life events, are crucial for connecting genotypes to clinical outcomes.

Several SNPs in SLC6A2 have been studied for their effects on NET function and expression [12]. Five well‐characterized SNPs include rs2242446, rs168924, rs2397771, rs5564, and rs5569 [13, 14, 15]. The rs2242446 polymorphism (T‐182C) resides in the 5′‐flanking promoter region of the NET gene, where cis‐elements crucial for transcription regulation are located. Changes in this region could affect transcriptional activity [13]. Similarly, the rs5569 polymorphism (G1287A) leads to a Gly392Arg substitution and is associated with altered NET expression, potentially increasing susceptibility to major depressive disorder [14]. Given NET's role in autonomic regulation, SLC6A2 polymorphisms may also influence VVS susceptibility in children.

Recent studies have broadened the understanding of how genetic factors, including SLC6A2 polymorphisms, contribute to VVS. Other genetic factors, such as endothelin‐1 polymorphisms and beta‐1 receptor gene variants (e.g., Arg389), suggest a wider genetic basis for syncope, particularly under orthostatic stress [16, 17]. Genetic and environmental interactions in children may differ significantly from adults, highlighting the need for targeted research. However, the role of SLC6A2 SNPs in pediatric VVS remains underexplored.

This study aims to investigate the association between five specific SLC6A2 polymorphisms and VVS risk in children. By focusing on well‐characterized SNPs and using rigorous diagnostic criteria, we aim to elucidate the genetic mechanisms underlying VVS in pediatric populations. The findings may offer new insights into VVS pathogenesis, identify potential therapeutic targets, and improve management strategies to reduce the frequency and impact of syncopal episodes in children.

## 2 Materials and Methods

### 2.1 Subjects

From June 2022 to December 2023, this study involved 142 children, including 71 VVS patients from the Department of Pediatrics at Qilu Hospital, Shandong University, and 71 age‐matched controls from the hospital's Health Examination Center. The study received approval from the Institutional Ethics Committee of Qilu Hospital of Shandong University (Approval No. KYLL‐202204‐065). Table 1 presents the characteristics of the research groups. Inclusion criteria for the VVS group were: (1) age under 18; (2) a history of at least two syncope episodes, one within the past 6 months; and (3) a positive Head‐up Tilt Test (HUTT). Exclusion criteria were: (1) cardiogenic conditions (e.g., cardiomyopathy, pulmonary embolism); (2) neurogenic conditions (e.g., seizure disorders, complex migraine); (3) metabolic disorders (e.g., hypoglycemia, hypocapnia); and (4) any systemic diseases (inflammatory, infectious, immunological, or nutritional) within 1 year before recruitment.

**Table 1:** Comparisons of demographic date and hemodynamic data between VVS cases and controls.

Variables	VVS	Control	*t*/*χ*²	*p*
Gender (male/female)	27/44	29/42	0.12	0.73
Age (years)	11.49 ± 2.31	12.07 ± 2.54	1.42	0.16
Underweight	3	1	1.03	0.31
Family histroy	8	1	4.27	0.04
Supine SBP (mmHg)	115.3 ± 7.09	114.6 ± 6.04	0.64	0.53
Supine DBP (mmHg)	68.89 ± 7.19	69.21 ± 6.44	0.28	0.78
Supine HR (beats/min)	74.28 ± 6.19	73.26 ± 6.13	0.97	0.34

For the control group, the inclusion criteria were: (1) children undergoing routine health examinations with no familial ties to the study subjects, and (2) no history of syncope. The same exclusion criteria applied to both groups.

Written informed consent was attained from parents or custodians. General clinical data included gender, family history, age, body mass index (BMI), supine systolic blood pressure (SBP), supine diastolic blood pressure (DBP), and supine heart rate (HR). Laboratory indicators included serum total cholesterol (TC), triglycerides (TG), Vitamin D, red blood cells (RBC), hemoglobin (HB), mean corpuscular volume (MCV), mean corpuscular hemoglobin (MCH) and platelets (PLT). BMI was categorized as underweight if less than the 5th percentile for age and gender.

### 2.2 Head‐Up Tilt Test (HUTT) Protocol

Medications affecting autonomic nervous function were discontinued for at least five half‐lives before testing. The child fasted for at least 4 h before the procedure, which was conducted in a quiet, dimly lit, temperature‐controlled environment. After lying supine for 10 min, baseline blood pressure and heart rate were recorded, with the average values used as baselines. The tilt table was then raised to a 60° head‐up position, and dynamic measurements of heart rate, blood pressure, and electrocardiogram (ECG) changes were recorded. Clinical symptoms were documented, and positive responses were assessed immediately. If no symptoms appeared after 45 min of tilting, sublingual glyceryl trinitrate spray (4–6 µg/kg) was administered, and the patient stayed head‐up for 10 more minutes. A positive HUTT result was defined by syncope or presyncope accompanied by one or more of the following: systolic blood pressure of 80 mmHg or lower, diastolic blood pressure of 50 mmHg or lower, and a reduction in mean arterial pressure exceeding 25%, bradycardia with age‐specific thresholds, sinus arrest, junctional escape rhythm or a brief atrioventricular block at second degree or higher.

### 2.3 SLC6A2 Gene Polymorphism Analysis

A 3 mL venous blood sample was drawn into EDTA tubes, and genomic DNA was isolated using the TIANGEN Blood Genomic DNA Extraction Kit. Genotyping for SLC6A2 SNPs (rs2242446, rs168924, rs2397771, rs5564, and rs5569) was carried out following the supplier's guidelines. The PCR reaction mixture included 0.5 μg of genomic DNA, 1 μL of forward primers and 1 μL reverse primers, and 16 μL of ultrapure water, 25 μL of 2× PCR Mix, adjusting the complete volume to 50 μL. PCR conditions were as follows: 95°C for 15 min, 50 cycles consisting of 30 s at 95°C, 30 s at 58°C, and 72°C for 5 min. Amplified fragments were sequenced on an ABI 3730XL DNA Sequencer (Applied Biosystems, USA).

### 2.4 Biochemical Analysis of Serum Samples

Blood specimens were obtained from the participants in the research after an overnight fast. Vitamin D level is evaluated by measuring the serum level of 25‐hydroxyvitamin D (25(OH)D) [18]. In this study, the measurement of 25(OH)D was performed using the Elecsys Vitamin D total III (Roche, Switzerland). The sample undergoes initial incubation with pretreatment reagents, followed by the addition of ruthenium‐labeled vitamin D binding protein (VDBP). Subsequently, streptavidin‐coated microparticles and biotinylated 25(OH)D are added, facilitating the formation of a complex between the ruthenylated VDBP and biotinylated 25(OH)D. The resulting mixture is then transferred into the measuring cell, where a voltage applied to the electrode triggers chemiluminescent emission, which is quantified using a photomultiplier.

HB, RBC, MCH, PLT, and MCV were obtained using the automated haematology analyser (XN‐9000, Sysmex, Japan). TC and TG were measured on an automated modular analyzer (Cobas 8000, Roche, Switzerland).

### 2.5 Statistical Analysis

Data were analyzed using SPSS 27.0 (SPSS Inc., Chicago, IL, USA). Genotype and allele frequencies were assessed for Hardy‐Weinberg equilibrium. Continuous variables were summarized as mean ± standard deviation (*x̄* ± sd). For variables that follow a normal distribution, independent samples *t*‐tests were used for group comparisons. For variables that do not follow a normal distribution, the Mann–Whitney *U* test was used for group comparisons. Genotype and allele frequencies were compared using chi‐square (*χ*²) tests. Relationships among genetic variants, clinical variables, and hematological indices were evaluated using logistic regression model. Two‐sided *p* values of below 0.05 denoted statistical significance.

## 3 Results

### 3.1 Baseline Data

This study involved 71 children with VVS (27 males) and 71 healthy controls (29 males). A summary of the demographic and hemodynamic data for both groups is presented in Table 1. Compared to the control group, VVS patients were found to have a significantly higher incidence of a positive family history (8/63 vs. 1/70, *χ*² = 4.27, *p* = 0.04). In the VVS group, the average age was 11.49 years, compared to 12.07 years for the control group. There were no significant differences between the two groups in terms of age, gender distribution, underweight status, HR, SBP, or DBP. Hematological parameters are outlined in Table 2. Children diagnosed with VVS exhibited significantly lower vitamin D levels (15.33 ± 5.33 ng/mL vs. 17.44 ± 5.56 ng/mL, *p* = 0.02) and higher HB concentrations (129.15 ± 9.34 g/L vs. 126.56 ± 13.55 g/L, *p* = 0.03) compared with the control group. Additionally, the VVS group showed an elevated MCV (86.12 ± 4.36 fL vs. 83.48 ± 7.38 fL, *p* = 0.02). No statistically significant differences were detected in RBC count, MCH, PLT count, TC, or TG levels (*p* > 0.05).

**Table 2:** Distribution of biochemical indexes of VVS cases and controls.

Variables	VVS	Control	*p*
Vitamin D (ng/mL)	15.33 ± 5.33	17.44 ± 5.56	0.02
HB (g/L)	129.15 ± 9.34	126.56 ± 13.55	0.03
RBC (×10^12^/L)	4.55 ± 0.32	4.56 ± 0.43	0.70
MCH (pg)	28.43 ± 1.66	27.85 ± 2.69	0.12
PLT (×10^9^/L)	266.92 ± 48.06	310.48 ± 117.33	0.08
MCV (fL)	86.12 ± 4.36	83.48 ± 7.38	0.02
TC (mmol/L)	3.66 ± 0.53	3.68 ± 0.58	0.78
TG (mmol/L)	1.031 ± 0.31	1.129 ± 0.33	0.07

### 3.2 Hardy‐Weinberg Equilibrium (HWE) Test

The genotype distribution of the SLC6A2 gene in both the patient and control groups is shown in Table 3. The genotype frequencies for both groups were consistent with Hardy–Weinberg equilibrium.

**Table 3:** HWE information for SLC6A2 gene polymorphisms in the VVS group and the control group with *χ* ^2^ results of the HWE testing.

Polymorphism	Genotype	VVS			Control		
		Number of patients	*χ* ^2^	*p*	Number of Patients	*χ* ^2^	*p*
rs2242446 (C > T)	CC	19	2.31	0.13	6	0.91	0.34
	CT	29			35		
	TT	23			30		
rs168924 (A > G)	AA	44	0.01	0.90	40	2.48	0.12
	AG	24			23		
	GG	3			8		
rs2397771 (C > G)	CC	4	2.95	0.09	8	0.43	0.51
	CG	37			28		
	GG	30			35		
rs5564 (A > G)	AA	30	0.19	0.66	44	2.08	0.15
	AG	31			21		
	GG	10			6		
rs5569 (G > A)	GG	38	0.003	0.96	33	0.09	0.77
	GA	28			30		
	AA	5			8		

### 3.3 Genetic Analysis of SLC6A2 Polymorphism

The association between the five SLC6A2 genotypes (rs2242446, rs168924, rs2397771, rs5564, and rs5569) and VVS was evaluated and compared using chi‐square (*χ*²) tests. Significant differences in genotype and allele distribution were found for rs2242446 and rs5564 between groups. The TT genotype exhibited a significant correlation with a higher risk of VVS when compared to the CC + CT genotypes of the rs2242446 polymorphism (CC + CT/TT, 31/40 vs. 45/26, *χ* ^2^ = 5.55, *p* = 0.02). For the rs5564 polymorphism, when comparing the AA genotype with the AG + GG genotypes, a significant correlation with an increased risk of VVS was also observed (GG + AG/AA, 39/32 vs. 27/44, *χ* ^2^ = 4.08, *p* = 0.04). No significant associations were found for rs168924, rs2397771, or rs5569 polymorphisms (*p* > 0.05, Table 4).

**Table 4:** Distribution of SLC6A2 genotypes in VVS cases and controls.

Polymorphism	Genotype	VVS (%)	Control (%)	*χ* ^2^	*p*
rs2242446 (C > T)	CC	7 (9.86)	10 (14.08)	5.55	0.06
	CT	24 (33.80)	35 (49.30)		
	TT	40 (56.34)	26 (36.62)		
	(CT + TT) vs. CC	64 (90.14)	61 (85.92)	0.60	0.44
	(CC + CT) vs. TT	31 (43.66)	45 (63.38)	5.55	0.02
	C	38 (26.76)	55 (38.73)	4.62	0.03
	T	104 (73.24)	87 (61.27)		
rs168924 (A > G)	AA	44 (61.97)	40 (56.34)	2.48	0.29
	AG	24 (33.80)	23 (32.39)		
	GG	3 (4.23)	8 (11.27)		
	(AA + AG) vs. GG	68 (95.77)	63 (88.73)	2.46	0.12
	(GG + AG) vs. AA	27 (38.03)	31 (43.66)	0.47	0.50
	A	112 (78.87)	103 (72.54)	1.55	0.21
	G	30 (21.13)	39 (27.46)		
rs2397771 (C > G)	CC	4 (5.63)	8 (11.27)	2.96	0.23
	CG	37 (52.11)	28 (39.44)		
	GG	30 (42.25)	35 (49.30)		
	(CC + CG) vs. GG	41 (57.75)	36 (50.70)	0.71	0.40
	(GG + CG) vs. CC	67 (94.37)	63 (88.73)	1.46	0.23
	C	45 (31.69)	44 (30.99)	0.02	0.90
	G	97 (68.31)	98 (69.01)		
rs5564 (A > G)	AA	32 (45.07)	44 (61.97)	4.18	0.12
	AG	29(40.85)	21 (29.58)		
	GG	10 (14.08)	6 (8.45)		
	(AA + AG) vs. GG	61 (85.92)	65 (91.55)	1.13	0.29
	(GG + AG) vs. AA	39(54.93)	27 (38.03)	4.08	0.04
	A	93 (65.49)	109 (76.76)	4.39	0.04
	G	49 (34.51)	33 (23.24)		
rs5569 (G > A)	GG	38 (53.52)	33 (46.48)	1.11	0.57
	GA	28 (39.44)	30 (42.25)		
	AA	5 (7.04)	8 (11.27)		
	(AA + AG) vs. GG	33 (46.48)	38 (53.52)	0.70	0.40
	(GG + AG) vs. AA	66 (92.96)	63 (88.73)	0.76	0.38
	G	104 (73.24)	96 (67.61)	1.08	0.30
	A	38 (26.76)	46 (32.39)		

### 3.4 Multivariate Analysis

Family history, age, vitamin D, TG, HB, PLT, MCH, MCV, and the rs2242446 and rs5564 polymorphisms were included in a logistic regression model to assess the combined impact of genetic and clinical factors on VVS susceptibility (Table 5). Family history (OR = 11.98, 95% CI = 1.18–121.54, *p* = 0.036), vitamin D (OR = 0.93, 95% CI = 0.86– > 0.99, *p* = 0.048), PLT (OR = 0.99, 95% CI = 0.99–> 0.99, *p* = 0.02), MCH (OR = 0.35, 95% CI = 0.18–0.70, *p* = 0.03), MCV (OR = 1.45, 95% CI = 1.14–1.84, *p* = 0.003), and rs2242446 (CC + CT vs. TT, OR = 2.54, 95% CI = 1.12–5.75, *p* = 0.03) were correlated with increased VVS risk. There was no significant association between TG levels, age, HB, or the rs5564 polymorphism and the occurrence of VVS (*p* > 0.05).

**Table 5:** Multivariate analysis of rs2242446, rs5564, and baseline factors in the research subjects.

Variables	*β*	*p*	OR	95% CI for OR	
				Lower	Upper
Family histroy	2.48	0.04	11.98	1.18	121.54
Age	−0.01	0.86	0.99	0.84	1.16
Vitamin D (ng/mL)	−0.08	0.05	0.93	0.86	> 0.99
TG (mmol/L)	−1.03	0.10	0.36	0.10	1.24
HB (g/L)	0.04	0.08	1.04	> 0.99	1.08
PLT (×10^9^/L)	−0.007	0.02	0.99	0.99	> 0.99
MCH (pg)	−1.04	0.003	0.35	0.18	0.70
MCV (fl)	0.37	0.003	1.45	1.14	1.84
rs2242446	0.93	0.03	2.54	1.12	5.75
rs5564	0.61	0.15	1.83	0.80	4.17

## 4 Discussion

Previous studies have linked VVS to various factors, including genetic predispositions and environmental influences. However, not all high‐risk individuals experience syncope, suggesting variability in susceptibility [19]. Recent evidence indicates that certain demographic groups exhibit a higher prevalence of VVS, showing clustering patterns similar to those observed in other conditions [20]. We hypothesize that individuals with frequent syncope episodes may have a genetic predisposition to VVS. The SLC6A2 gene encodes NET, which has been recognized as a potential therapeutic target for the treatment of VVS [5, 21]. Polymorphisms in SLC6A2 have been associated with differential autonomic responses [22]. Given its role in autonomic regulation, we hypothesized that SLC6A2 polymorphisms contribute to VVS risk. To test this, we conducted a case‐control study examining the interaction between genetic polymorphisms and environmental factors. Participants were divided into VVS and control groups, and five SLC6A2 SNPs—rs2242446, rs168924, rs2397771, rs5564, and rs5569—were genotyped using TaqMan assays. Our findings suggest that genetic screening for SLC6A2 variations may enhance syncope diagnosis.

In this study, our preliminary analysis identified potential associations between the SLC6A2 gene polymorphisms rs2242446 and rs5564 and VVS susceptibility in children. The frequency of the rs2242446 TT genotype was significantly elevated in the VVS group relative to controls (56.34% vs. 36.62%, *p* = 0.02). Similarly, the VVS group had a higher frequency of the GG and AG genotypes of rs5564 compared to controls (54.93% vs. 38.03%, *p* = 0.04). No associations were detected for the rs168924, rs2397771, and rs5569 polymorphisms with VVS risk. We further examined the combined effects of gene polymorphisms and baseline characteristics on VVS etiology. Analysis showed correlations between VVS and vitamin D deficiency, family history, HB, and MCV (*p* < 0.05). Multivariate analysis included baseline factors and genotype data from rs2242446 and rs5564. Among the SLC6A2 polymorphisms, only rs2242446 was significantly associated with VVS. Other variant, rs5564, did not significantly contribute to VVS risk when combined with environmental factors. Notably, our results indicated that vitamin D deficiency increased susceptibility to VVS, corroborating findings from a 2023 systematic review and meta‐analysis, which showed a higher incidence of VVS in vitamin D‐Deficient patients [23]. Although no direct causal relationships were established between VVS and hematological parameters such as PLT, MCH, and MCV, these factors may indirectly influence syncope risk by affecting overall health and circulatory dynamics [24, 25].

This study establishes a significant correlation between the rs2242446 polymorphism in the SLC6A2 gene and an elevated risk of VVS in pediatric populations. These findings provide valuable insights into the genetic mechanisms underlying VVS and contribute to our understanding of its pathogenesis. The SLC6A2 gene, located on chromosome 16, consists of multiple exons and introns [24]. It encodes the NET, a key protein in the sympathetic nervous system responsible for regulating norepinephrine reuptake. This process influences the duration and intensity of norepinephrine's effects on target organs, including the heart and blood vessels [25]. A polymorphism in the SLC6A2 gene, such as rs2242446, may affect NET function or expression, potentially impairing norepinephrine reuptake. This dysregulation of sympathetic activity could disrupt blood pressure and heart rate regulation, thereby increasing the risk of VVS.

The rs2242446 polymorphism in the SLC6A2 gene, located in its promoter region, plays a crucial role in the regulation of the NET. This polymorphism, specifically the T allele, has been consistently linked to an increased risk of suicide in patients with major depressive disorder (MDD) across various studies [6]. The T allele appears to influence gene expression levels, potentially affecting NET function and subsequently altering norepinephrine availability in synapses, which may exacerbate depressive symptoms [13]. Moreover, the rs2242446 polymorphism has been associated with specific symptom clusters in psychiatric conditions. For instance, in posttraumatic stress disorder (PTSD), individuals carrying the T allele exhibit heightened anxious arousal, characterized by increased hypervigilance and exaggerated startle responses [26]. This suggests that rs2242446 may modulate norepinephrine clearance, thereby influencing the severity and nature of psychiatric symptoms. Despite being located in a noncoding region, the regulatory effects of this SNP have drawn considerable attention due to its potential role in modulating gene expression and contributing to disease pathogenesis in neuropsychiatric disorders [27]. As a result, rs2242446 represents a promising target for further research into genetic markers of psychiatric risk and treatment outcomes.

In contrast to rs2242446, other loci within the SLC6A2 gene did not show significant associations with VVS, possibly due to their minimal impact on NET function or a lack of involvement in the primary pathogenesis of VVS. Additionally, VVS is likely influenced by multiple genetic and environmental factors, suggesting that individual loci within SLC6A2 may not fully account for its genetic background. The multifactorial nature of VVS implies that while rs2242446 is a significant risk factor, it is not the sole determinant of susceptibility.

These findings are consistent with previous research implicating the norepinephrine system in VVS pathogenesis [5, 28]. Prior studies have reported abnormalities in norepinephrine levels and NET function in VVS patients, supporting the hypothesis that autonomic nervous system dysregulation is central to syncope development [29]. However, this study is the first to identify a specific genetic locus within SLC6A2 (rs2242446) directly associated with VVS, offering deeper insights into the genetic factors that contribute to this condition.

This study had several limitations. First, the sample predominantly consisted of individuals from a specific geographic region and ethnic background, potentially limiting the generalizability of the findings. The association between rs2242446 and VVS may vary across populations, necessitating replication studies in more diverse cohorts to validate these results. Additionally, the observational design precludes definitive conclusions about causality. While the association between the rs2242446 polymorphism and VVS is statistically significant, in the absence of additional molecular evidence, this study does not aim to clarify the pathophysiological mechanisms. Future studies are warranted to clarify the molecular pathways. Furthermore, this study focused on a pediatric population, and it would be valuable to investigate whether the association holds true in adults. Since VVS can affect individuals across the lifespan, exploring how genetic risk factors like rs2242446 may vary with age is a crucial area for future research.

## 5 Conclusion

In conclusion, this study identifies a significant association between the rs2242446 polymorphism in the SLC6A2 gene and the risk of VVS in children, providing new insights into the genetic factors contributing to this condition. These findings represent an important step forward in understanding the genetic architecture of VVS and suggest potential avenues for developing genetic screening tools and targeted interventions. As research builds on these results, the ultimate goal is to translate genetic insights into clinical practice, improving our ability to predict, prevent, and treat VVS.

## Author Contributions

**Minmin Wang:** writing – original draft, data curation, formal analysis. **Meng Li:** conceptualization, data curation, formal analysis. **Haizhao Zhao:** writing – review and editing. **Xiaoyue Liu:** investigation, writing – review and editing. **Qingyu Kong:** funding acquisition, writing – review and editing. **Cuifen Zhao:** validation, methodology, supervision, resources. **Baomin Li:** writing – review and editing; conceptualization.

## Ethics Statement

This study was approved by the Institutional Ethics Committee of Qilu Hospital of Shandong University (Approval No. KYLL‐202204‐065).

## Consent

Before being recruited, all eligible participants provided informed consent and assent.

## Conflicts of Interest

The authors declare no conflicts of interest.

## Transparency Statement

The lead author Qingyu Kong, Cuifen Zhao, Baomin Li affirms that this manuscript is an honest, accurate, and transparent account of the study being reported; that no important aspects of the study have been omitted; and that any discrepancies from the study as planned (and, if relevant, registered) have been explained.

## Data Availability

The data that support the findings of this study are available on request from the corresponding author. The data are not publicly available due to privacy or ethical restrictions.
